# Malondialdehyde Adduct to Hemoglobin: A New Marker of Oxidative Stress Suitable for Full-Term and Preterm Neonates

**DOI:** 10.1155/2013/694014

**Published:** 2013-06-13

**Authors:** Cécile Cipierre, Stéphane Haÿs, Delphine Maucort-Boulch, Jean-Paul Steghens, Jean-Charles Picaud

**Affiliations:** ^1^Département de Néonatologie, CHU Angers, 4 rue Larrey, 49100 Angers, France; ^2^Département de Néonatologie, Hôpital de la Croix Rousse, Hospices Civils de Lyon, 69004 Lyon, France; ^3^Centre de Recherche en Nutrition Humaine Rhône-Alpes, Centre Hospitalier Lyon-Sud, 69310 Pierre Bénite, France; ^4^Département de Biostatistique, Hospices Civils de Lyon, 69003 Lyon, France; ^5^Université Claude Bernard Lyon I, 69100 Villeurbanne, France; ^6^CNRS UMR5558, Laboratoire de Biométrie et Biologie Evolutive, Equipe Biostatistique Santé, 69310 Piere-Bénite, France; ^7^Centre de Biologie Sud, UF Nutrition et Métabolisme, Centre Hospitalier Lyon-Sud, Hospices Civils de Lyon, 69310 Pierre Bénite, France

## Abstract

Oxidative stress may play a central role in the onset of many diseases during the neonatal period. Malondialdehyde (MDA) is a marker of lipid peroxidation. The aim of this study was to evaluate a new marker, the malondialdehyde adduct to hemoglobin (MDA-Hb), which is measured in red blood cells (RBCs) and thus does not require that an additional blood sample be drawn. In this prospective study, we first adapted the measurement method previously described to Hb solutions obtained from washed RBCs and then evaluated the suitability of the method for use in neonates. MDA-Hb concentrations were measured by liquid chromatography-mass spectrometry. We compared the concentrations of MDA-Hb between preterm and term neonates. Erythrocyte samples were collected at birth from 60 healthy neonates (29 full-term and 31 preterm), as well as from 50 preterm neonates with uncomplicated postnatal evolution during the first months of life. We found a significantly higher MDA-Hb concentration at birth in preterm neonates (*P* = 0.002). During the first months of life, MDA-Hb concentrations were 9.4 nanomol/g Hb in hospitalized preterm neonates. MDA-Hb could be used to assess oxidative stress in preterm neonates. Together with clinical variables, it could be a useful marker for oxidative stress exposition in these higher risk patients.

## 1. Introduction

Growing evidence indicates that an imbalance between oxidative stress (OS) and antioxidant defense mechanisms plays an important role in the onset of many diseases during the neonatal period [1, 2]. Birth is associated with a strong OS related to the rapid change from a relatively hypoxic intrauterine environment to the extrauterine environment (5-fold increase in alveolar PO_2_) and several physiologic processes involved in the delivery [3, 4]. These changes and processes greatly increase the production of free radicals, which must be controlled by the antioxidant defense system that has been maturing over the course of gestation [5, 6].

Free radicals are too short-lived to be detected directly in clinical systems, but oxygen free radicals react with lipids to produce lipid peroxidation products, which when measured serve as indirect biomarkers of *in vivo *oxidative stress status and related diseases. Among these products, malondialdehyde (MDA) is one of the principal and most studied low-molecular-weight end products. It is highly cytotoxic because of its ability to bind proteins or nucleic acids very quickly [7].

The thiobarbituric acid reactive substance method (TBAR test) has been frequently used to assess MDA concentrations, but it lacks specificity as aldehydes other than MDA (formaldehyde, acetaldehyde, etc.) and non lipid substances react with thiobarbituric acid [8, 9]. Other analytical techniques such as specific derivatization before liquid chromatography with UV or mass spectrometry detection have been proposed to measure MDA more precisely [10]. It should be noted that all these techniques reflect measures only at a specific moment, although peroxidation reactions fluctuate over time. 

Determining adducts to proteins, particularly Hb, has proved to be a useful approach for monitoring *in vivo* exposure to genotoxic compounds [11]. An adduct is formed when a low molecular compound binds with a biological molecule [11]. The Hb adducts are stable reaction products derived from electrophilic compounds, by covalent attachment, involving nucleophilic centers in biomolecules that offer possibilities for the sampling and analysis of electrophilic, short-lived compounds [12].

The determination of MDA-Hb obtained with a very complex method has thus been proposed to assess the exposure to lipid peroxidation products [13]. In healthy adults, MDA-Hb values of 0.01 to 10 nanomol/g Hb were reported [14]. Moreover, the method used in these studies was sensitive enough to detect variations in the levels of MDA adducts caused by lipid peroxidation, with a direct correlation between dose and effect [15].

MDA-Hb measurement could provide an assessment of OS in neonates over the middle term because of (i) the stable covalent attachment between MDA and Hb and (ii) MDA-Hb elimination, which is dependent on the relatively well-defined life span of the erythrocyte (120 days in human adults, shorter in neonates) [16]. We tested whether we would be able to determine MDA-Hb in neonates without taking more blood from them, as this is a crucial issue in very low birth weight (VLBW) neonates. We assumed that the erythrocytes remaining from routine blood samples taken for electrolyte determination would be sufficient.

We thus evaluated the feasibility of MDA-Hb measurement in neonates and sought to establish normal MDA-Hb ranges in healthy full-term neonates at birth and uncomplicated premature neonates at birth and during the first months of life. 

## 2. Methods

### 2.1. Study Populations

To assess MDA-Hb at birth, neonates born on the maternity ward of Croix Rousse University Hospital, Lyon, France, were consecutively enrolled between February and May 2009. They were divided in two groups according to their gestational age (GA). In the first group, inclusion criteria were: healthy neonates born after a full-term gestation (≥37 weeks), delivered vaginally without complications, and presenting good adaptation to extrauterine life (no resuscitation, no evidence of perinatal hypoxia, or respiratory distress), birth weight (BW) appropriate for GA [17], and a normal clinical examination at birth. In the second group, inclusion criteria were: premature neonates (28–36 weeks) who did not require intensive care (no intubation, no chest compression, or drugs for resuscitation), oxygen therapy, or any type of medication at birth. Exclusion criteria were as follows: the need for resuscitation, evidence of perinatal hypoxia (pH ≤ 7.20 in cord blood, 5 min Apgar score <7) or respiratory distress, congenital malformation, sepsis, and small for GA [17], and multiple gestation. Measurements of MDA-Hb at birth were performed in 29 full-term and 31 premature neonates. 

To measure MDA-Hb in the first months of life, we collected blood samples from uncomplicated premature neonates, that is, without assisted ventilation, parenteral nutrition, blood transfusion, or anti-inflammatory treatment within the five days before blood sampling. We recorded BW, GA, gender, and postnatal age on the day of sample collection. Measurements of MDA-Hb during the first 8 weeks of life were performed in 50 uncomplicated preterm neonates as previously defined.

The study was completely integrated into the routine care of full-term and preterm neonates and there was no supplementary blood sampling required. All parents signed an informed consent form. The study was approved by the Ethics Committee of the University Hospital Center of Lyon, France, (*CPP Lyon Sud Est IV*). 

### 2.2. Determination of MDA-Hb

#### 2.2.1. Collection of Samples

The assessment of MDA-Hb required the collection of erythrocytes. At the time of birth, arterial cord blood (5 mL) was collected from full-term and preterm neonates who fulfilled the inclusion criteria. As cord blood pH is systematically assessed at birth in our hospital, blood samples for the purpose of our study were also obtained at that time.

During the hospitalization of the preterm neonates who fulfilled the inclusion criteria, erythrocyte samples were collected at the same time as the venous blood sampling usually required for routine assessment of serum electrolytes in these neonates. According to the routine procedure, serum and red blood cells were separated by centrifugation (10 minutes, 4000 G) in the hospital biochemistry laboratory. The tubes were then immediately refrigerated at 4°C until the preparation of samples.

#### 2.2.2. Reagents

Malondialdehyde-bis-dimethylacetal (tetramethoxypropane, TMP) 99% purity and metaphosphoric acid were purchased from sigma- Aldrich Chemie GmbH (Steinheim, Germany). Dideuterated tetraethoxypropane was from CDN Isotopes (88 Leacock Street, Pointe-Claire, QC, Canada). 2,3-Diaminonaphthalene (DAN) was obtained from TCI. Anhydrous potassium dihydrogen phosphate (Suprapur), ethanol, methanol, and acetonitrile gradient grade were from Merck (Darmstadt, Germany). All other chemicals and solvents were of analytical grade. Aqueous solutions were made with pure water (conductivity ≥18 MΩ), Elga Pure Lab Option (Elga, France). 

Standard stock solution of TMP 608 nM was obtained by seven successive dilutions (1/10) of TMP: three times in ethanol then three times in ethanol/water (40/60, v/v) and finally in water with NH_4_OH 90 mM final concentration.

Aliquots of this solution are stable under nitrogen for 3 months at −20°C in the dark. Secondary MDA standards (76, 152, and 304 nM) were prepared daily by dilution in water of the standard stock solution.

#### 2.2.3. Material

LC-MS analysis was carried out on an Agilent LC 1100 series chromatograph with an Agilent LC-MSD SL single quadrupole as detector. The chromatographic mobile phase was ammonium acetate 5 mmol/L, adjusted at pH 1.8 with formic acid containing 15% (v/v) of a methanol-acetonitrile (1 : 1) mix. Derivatized samples and standards (10 *μ*L) were injected on a 150∗2 mm Uptishere HDO C18 (3 *μ*m particle size) column (Interchim, Montluçon, France). The flow rate was 0.23 mL/min with the column kept at 50°C in a column oven. 

The quantification was carried out with a dideuterated MDA (d2-MDA) internal standard, and the derivatives of MDA and d2-MDA were detected in ESI positive mode (M + H)^+^ at *m*/*z* 195.2 and 197.2, respectively.

#### 2.2.4. Method

The procedure for measuring MDA-Hb consisted of three steps:

- isolation of Hb and delipidation in order to avoid any artifactual lipid peroxidation,
- hydrolysis and derivatization of the MDA adduct with DAN to form a diazepinium complex,
- then quantification of the diazepinium by LC-MS.

After a first centrifugation, RBCs (200 *μ*L) were washed two times with four volumes of NaCl (9 g/1000 mL), and centrifuged (5 minutes, 1000 G). 150 *μ*L of washed and packed RBCs were resuspended in distilled water (450 *μ*L) and freeze-dried at −80°C for 5 minutes then thawed in hot water (30 seconds under water at 60°C). After a second cycle of freeze drying-thawing, Hb solution was obtained by centrifugation for 4 min at 8000 G; one aliquot was used to measure Hb concentration and another aliquot (200 *μ*L) was rapidly delipidated by mixing with 100 *μ*L Folch reagent (methanol/chloroform, 1 vol./2 vol.) and centrifuged 5 minutes at 13000 G. The delipidated Hb from the top phase was either hydrolyzed and derivatized with DAN or stored at −20°C until analysis. 

Hydrolysis and derivatization, modified from the original method described for free and bound plasmatic MDA, were done according to Table 1. Preliminary experiments showed that to decrease adsorption of the diazepinium (formed between MDA and DAN) to Hb, derivatization had to be done in NaCl (60 g/L). 

The adduct of MDA to Hb was expressed in nanomol per gram Hb (nanomol/g Hb).

#### 2.2.5. Reproducibility of Sample Processing

The sample processing (RBCs washing, hemolysis, and delipidation) was checked on two series of measurements with four and six samples, respectively. Each sample was processed four or five times, depending on the available sample volume.

#### 2.2.6. Processed Sample Stability

Twenty-four samples were fully prepared, aliquoted, and stored at −20°C to check the stability of the processed samples. The aliquots were analyzed after 7, 20, and 32 days of storage.

### 2.3. Statistical Analysis

Categorical variables are expressed as numbers and percentages, whereas continuous variables are expressed as medians and ranges. All variables are described for the whole population and each group: full-term (≥37 weeks) and preterm (<37 weeks) neonates. We compared the characteristics (GA, BW, and gender) of the full-term and preterm neonates. Categorical variables were compared using the *χ* ^2^ test, and MDA-Hb concentrations were compared with the Mann-Whitney test. Correlation coefficients between MDA-Hb concentration and GA, BW, and gender were tested with the Spearman test. All tests were considered to be significant for *P* values less than 5%. Analyses were performed using SPSS, version 15.0 (Statistical Product and Service Solutions 15.0; SPSS Inc., Chicago, Il USA).

## 3. Results

### 3.1. Optimization of Derivatization Conditions

The main modification for the sample derivatization, done according to Table 1, was that all the reagents used were prepared in NaCl (60 g/L) to decrease the adsorption of the formed diazepinium to Hb. 

### 3.2. LC-MS Separation and Repeatability

**Figure 1:**
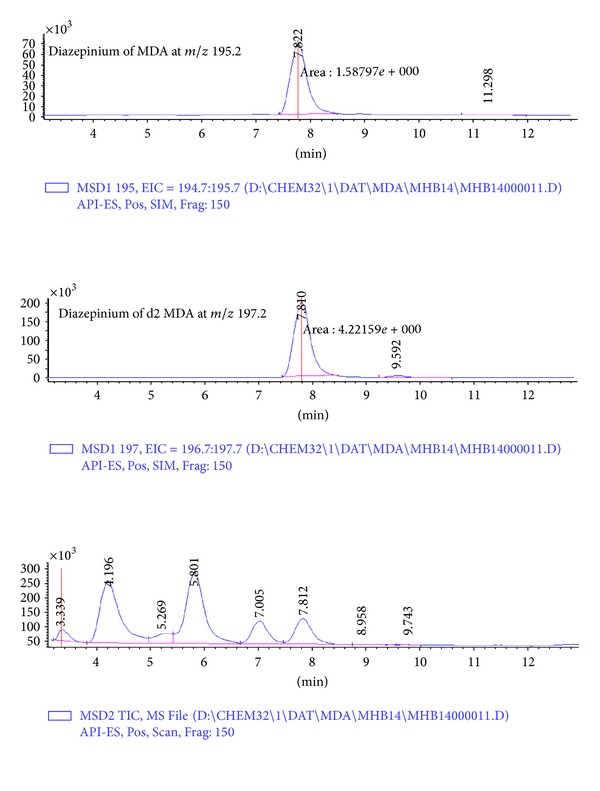
A typical chromatogram for the measurement of malondialdehyde adduct to hemoglobin (MDA-Hb). The upper trace is that of the diazepinium of MDA with its corresponding internal standard in the middle trace. The lower trace is that of the chromatogram in the total ion current (TIC) mode with a scan between *m*/*z* 100 and 300.

Figure 1 shows a typical chromatogram of the MDA adduct measured as a diazepinium at *m*/*z* 195.2 and the corresponding internal standard at *m*/*z* 197.2. The method was found to be linear up to 1000 nM. The repeatability of ten successive injections of the same sample was better than 95% (personal data).

### 3.3. Reproducibility of Sample Processing

Even if it is known that handling of packed RBCs is tricky, unexpectedly, first experiments showed that final variation of the results came essentially from sample processing. After optimization and with a good practice, the coefficient of variation (CV) for sample processing evaluated by Hb measurement of delipidated samples (two series with 6 and 4 samples resp., measured four times each) was below 5%, except for one sample (CV = 5.98%).

### 3.4. Processed Sample Stability

**Figure 2:**
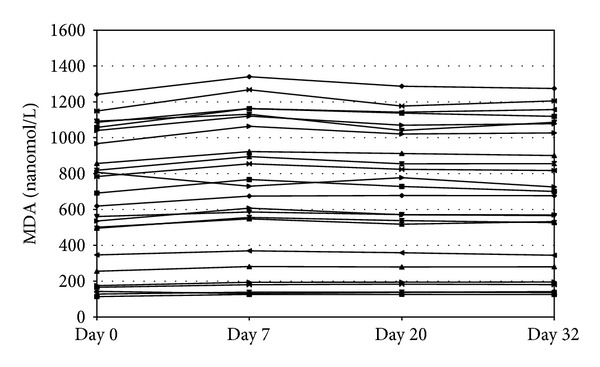
Processed sample stability of MDA: twenty-four different samples prepared, aliquoted, stored at –20°C, and analysed after 7, 20, and 32 days of storage.

Figure 2 shows the stability of 24 processed samples, aliquoted and stored at −20°C for at least a month. Aliquots were analyzed at day 0 and after 7, 20, and 32 days of storage.

### 3.5. Measurements of MDA-Hb

**Figure 3:**
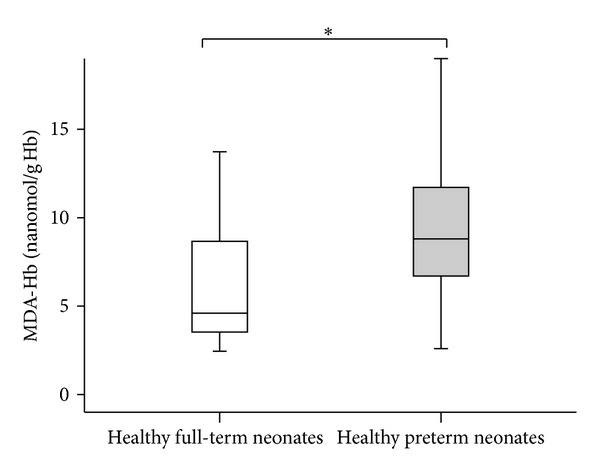
Concentrations of malondialdehyde adduct to hemoglobin (MDA-Hb) at birth in healthy full-term (*n* = 29) and healthy preterm neonates (*n* = 31). Values shown are median levels (25th/75th box; 10th/90th error bars). *Mann-Whitney test: significantly different from full-term neonates (*P* = 0.002).

The characteristics of the studied population are presented in Table 2. The MDA-Hb concentrations at birth were significantly higher in preterm neonates (8.8  (2.6–19.2) nanomol/g  Hb) than in full-term  neonates (4.6 (2.5–19.3) nanomol/g Hb) (*P* = 0.002) (Figure 3). 

**Figure 4:**
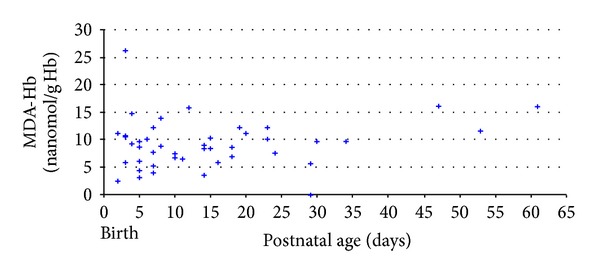
Relationship between concentrations of malondialdehyde adduct to hemoglobin (MDA-Hb) and postnatal age in healthy preterm neonates (*n* = 50).

MDA-Hb values during hospitalization were collected in preterm neonates (32.3  (26.5–35.5) weeks) with appropriate GA and BW (1495 (720–2720) g), and measurements were performed at a postnatal age of 12 (2–61)  days (Figure 4). During the first 8 weeks of life, 50 samples were analyzed in 50 uncomplicated preterm neonates (only one sample per preterm neonate) as previously defined. MDA-Hb concentrations were 9.4 (2.4–26.3) nanomol/g Hb. No correlation was found between BW or patient gender and the MDA-Hb concentration. 

At birth, GA was significantly and negatively correlated with MDA-Hb concentration (*r* = −0.31, *P* = 0.019). 

## 4. Discussion

We report a convenient method to determine the concentration of MDA adduct to Hb in neonates, which is sensitive enough to detect low concentrations of MDA-Hb and specific enough to assess lipid peroxidation.

The method to assess MDA-Hb is an adaptation of a reliable and validated method [10] used to evaluate the impact of parenteral nutrition composition on lipid peroxidation [18–20]. MDA-Hb was measured by LC-MS using a very specific method based on diaminonaphthalene derivatization [10]. The advantages of the method are its high sensitivity and specificity which rely on the use of DAN. When reacted with MDA, DAN forms a diazepinium with a mass of 194 Dalton higher than that of native MDA (72 Dalton) which makes its detection easier, because of an improved affinity for reverse phase column and this higher mass outside the background noise. A direct consequence of this high sensitivity is the low volume of sample needed for measurement (only 200 *μ*L). This is particularly interesting for neonatal care. Typical intra-assay variability is good (5%), and good reproducibility was observed. Although this new method requires rigorous conditions for sample preparation and measurement and may lead to the formation of artifacts, it has the advantage of being relatively simple, which is important for clinical practice. Our results thus suggest that this method is a useful, sensitive, and specific assay for lipid peroxidation in neonates. 

Furthermore, this assay has several advantages from a clinical point of view, not the least of them being the simplicity of collecting a component of blood (erythrocytes), that is, usually discarded after serum has been taken for electrolyte assessment from routine blood sampling. The relatively long and well-controlled life span of Hb was an important reason for choosing this protein as a dose monitor for electrolytically reactive compounds. Hb has a predetermined life span equal to that of red blood cells. MDA-Hb could therefore be a long-term indicator of oxidative stress. Moreover, since proteins such as Hb are present in blood in much larger amounts, measurement of protein adducts favors a high capacity of detection [11]. 

We observed that the method to measure Hb adducts was sensitive enough to detect variations in MDA-Hb concentrations at different GAs at birth. As expected, we observed significantly higher MDA-Hb levels in preterm neonates than in full-term neonates. Indeed, increased oxidative stress in preterm neonates at birth has been described using other oxidative stress markers [4, 6, 21, 22].

Based on these preliminary results, it appears that this marker might be useful in a variety of pathological conditions, such as VLBW neonates, and thus, its clinical relevance should be validated in a larger population. The ability to detect lipid peroxidation noninvasively is of particular importance in preterm neonatal care. A noninvasive technique would be a great aid in improving knowledge about oxidative stress in these patients and evaluating future improvements in neonatal care (assisted ventilation, oxygen therapy, and parenteral nutrition) for diseases related to reactive oxygen species. 

In summary, our results suggest that *in vivo* assessment of MDA-Hb in neonates is a feasible, blood-sparing, and simple method to determine oxidative stress, notably in preterm neonates.

## Figures and Tables

**Table 1:** Derivatization of standards and samples.

	Standards (from 76 to 608 nM)	MDA-Hb
Sample volume	100 *μ*L TMP	100 *μ*L
Derivatization	100 *μ*L DAN 5.8 mM in HCl 2.4 N + internal standard	

Mixing, incubation for 180 min at 37°C		

Hb precipitation	100 *μ*L MPA 10%, mixing, centrifugation 3 min, 15000 × g	
	200 *μ*L supernatant for the next step	
Stabilization at pH 2.0*	100 *μ*L (NaOH 1 N + KH_2_PO_4_ 300 mM pH 2.0)	

*At every new preparation of both reagents, the volumes are adjusted to obtain pH 2.0 with a fixed volume of 300 *μ*L (DAN: diaminonaphthalene; MDA: malondialdehyde; MPA: metaphosphoric acid; TMP: tetramethoxypropane).

**Table 2:** Clinical characteristics (mean (range unless specified *n* (%))) of 60 full-term neonates (GA ≥ 37 weeks, *n* = 29) or preterm (GA < 37 weeks, *n* = 31).

	Full-term neonates	Preterm neonates	*P* value
Gestational age at birth, weeks	39.4 (37.4–41.6)	31.7 (28.1–35.7)	<0.001
Birth weight, grams	3380 (2830–4270)	1580 (730–2580)	<0.001
Male, *n* (%)	14 (48.3)	15 (48.4)	0.993

## Abbreviations

BW: Birth weight
DAN: Diaminonaphthalene
GA: Gestational age
Hb: Hemoglobin
LC-MS: Liquid chromatography-mass spectrometry
MDA: Malondialdehyde
MDA-Hb: Malondialdehyde adduct to hemoglobin
RBCs: Red blood cells
ROS: Reactive oxygen species
TMP: Tetramethoxypropane
OS: Oxidative stress
VLBW: Very low birth weight.
